# The economic impact of the switch from single- to multi-dose PCV13 vial in Benin

**DOI:** 10.1186/s12889-021-12108-6

**Published:** 2022-01-19

**Authors:** Patrick G. Ilboudo, Téné-Alima Essoh, Roch A. Houngnihin, Daleb Abdoulaye Alfa, Naomi Dick, Landry Kaucley, Alexis Satoulou-Maleyo

**Affiliations:** 1 Agence de Médecine Préventive, Ouagadougou, Burkina Faso; 2 Agence de Médecine Préventive, Bureau régional Afrique, Abidjan, Côte d’Ivoire; 3 Laboratoire d’Anthropologie Médicale Appliquée, Cotonou, Bénin; 4 Maladies Infectieuses et Vecteurs : Ecologie, Génétique, Evolution et Contrôle, Institut de Recherche pour le Développement, Centre National de la Recherche Scientifique, Cotonou, Bénin; 5 Agence Nationale pour la Vaccination et les Soins de Santé Primaires, Cotonou, Bénin; 6 Organisation Mondiale de la Santé, Ouagadougou, Burkina Faso

**Keywords:** Economic impact, Switch, PCV13, Single-dose, Multi-dose, Benin

## Abstract

**Background:**

Little is known on the economic implications of multi-dose 13 valent pneumococcal conjugate vaccine (PCV13) introduction in expanded program on immunization (EPI). Based on evidence of PCV13’s reduced pressure on vaccine cold chain, Benin, a third world country in West Africa, introduced the multi-dose PCV13 starting in April 2018 in its EPI program in replacement of the single-dose presentation. The objective of this study was to conduct a rapid assessment of the costs and economic impact of switching from single- to multi-dose PCV13 vial in Benin.

**Methods:**

The data collected retrospectively between January 1 and February 16, 2019 using a quantitative questionnaire was analyzed using Excel 2010 and Stata 13. Resources consumed from April 1st to September 30th, 2017 for the single-dose PCV13 and from April 1st to September 30th, 2018 for multi-dose were analyzed. For both presentations, costs analyzed included vaccines, injections supplies, waste management, cold chain, personnel (salaries and per diems), supervision and monitoring, training, social mobilization and overheads. Moreover, additional costs incurred for the introduction of multi-dose PCV13 were also collected. Costs were estimated for each presentation of PCV13 vaccine by calculating the half-year value of recurrent and capital costs, discounted at a rate of 3% for capital items. To enable comparisons, costs pertaining to 2017 were converted to 2018 equivalent values taking inflation in US$ into account.

**Results:**

The economic costs of the single-dose PCV13 exceeded that of the multi-dose: US$ 3,708,795 versus US$ 3,698,795, respectively. Three cost items, including costs of vaccines, injection supplies, and cold chain appeared to be the main drivers of the observed reduction in costs of multi-dose PCV13. Moreover, the cost per infant vaccinated was lower with the single-dose PCV13 than the multi-dose, respectively US$ 6.28 versus US$ 10.92, and costs of vaccines wasted higher for the multi-dose PCV13.

**Conclusions:**

This evaluation seemed to show that the switch from single- to multi-dose PCV13 resulted in reduced economic costs of PCV13. Vaccinating more infants together with a rigorous application of vaccine open vial policy could lead to the change being more cost-effective.

**Supplementary Information:**

The online version contains supplementary material available at 10.1186/s12889-021-12108-6.

## Background

*Streptococcus pneumoniae* is one of the leading causes of severe pneumonia in adults, and bacterial meningitis in children less than 1 year [1]. Severe pneumococcal infections is also relatively important in children less than 5 years, with 18% of all severe pneumonia infections caused by *S. pneumoniae* [2]. Mortality from severe pneumococcal infections is also important [3] since 9 to 19% of Sp meningitis diagnosed in health facilities succumb to the infection [4, 5]. Furthermore, pneumococcal meningitis-related sequelae, including deafness, blindness and intellectual deficit, can be very serious, even disabling [6]. In addition, global statistics of under-five mortality indicated that a high proportion was due to pneumococcal infections [1].

The fight against pneumococcal infections includes vaccination which has been recognized as the most cost-effective strategy for reducing pneumococcal-related morbidity and mortality [7]. PCV13 is a pneumococcal conjugate vaccine providing protection against 13 serotypes of pneumococci [8]. The vaccine has been strongly recommended for young children [9], and adults at high risk of acquiring pneumococcal infections [10]. In 2010, Benin, with the support of The Global Alliance for Vaccines and Immunizations (GAVI), introduced the single-dose PCV13 vaccine in its EPI Program. In 2017, a multi-dose vial presentation (4-dose vial) has been made available on the market at a reduced cost per dose [11]. Upon its prequalification by WHO, and eligibility for GAVI support, Benin’s introduced the multi-dose PCV13 in its Expanded Program on Immunization (EPI) in April 2018. This introduction was prompted by the evidence suggesting a reduced pressure of the multi-dose PCV13 on vaccine cold chain, consisting of intertwined links that are designed to keep vaccines within WHO recommended temperature ranges, from the point of manufacture to the point of delivery. The introduction of new vaccines into EPI programs may consist in the addition of a new antigen against a disease not yet covered by the program. It also may consist in the introduction of a new formulation of a vaccine already present in the EPI program, or a combination vaccine replacing an old vaccine, or the replacement of a vaccine presentation by another presentation of the same vaccine.

The introduction of a new vaccine may induce a number of changes, including a new way for communicating for social mobilization (i.e. activities carried out to raise awareness of and demand for immunization including media and special events, advocacy, etc.), training health personnel on how to administrate the new vaccine, expanding the chain cold, etc. [12]. All these changes induce costs. However, little is known on the costs and impact of switching from single- to multi-dose PCV13. Data on the costs and economic impact of the switch from single- to multi-dose PCV13 are needed for decision-making on the introduction of this new presentation of the PCV13. The objective of this study was to conduct a rapid assessment of the costs and economic impact of the switch from single- to multi-dose PCV13 vial in Benin. Specifically, it aimed at comparing the costs and impact of PCV13 single- and multi-dose vaccines, basing on the recent introduction of the multi-dose PCV13 into Benin’s EPI.

## Methods

### Study setting

This study was conducted in Benin, a third world country located in West Africa, whose population was estimated at 11.9 million in 2019 [13]. The country’s economic growth potential is highly dependent on the agricultural sector, and poverty remains predominant. Slightly more than one in three inhabitants live below the subsistence minimum, and one in three still suffers many deprivations in terms of living conditions and wealth, with GDP per capita estimated at $901.50 in 2018 [14]. This widespread poverty limits women in infants’ access to education and healthcare, particularly in rural places. Infant mortality estimated at 61 per 1000 live births in 2018 [15] seemed to be partly explained by inadequate vaccination coverages. Complete vaccination coverage among infants was estimated to vary between 71 to 89% for MCV1, BCG, polio vaccine, DTP1, DTP3, and PCV13 in 2019 [16]. With respect to the studied period, coverages have been estimated at 73% for the 3rd dose of PCV13 in 2017 and 2018 [17]. Total and domestic government health expenditure per capita were $31 and $9, respectively in 2017 [18], and immunization spending per capita was $0.89 over 2005–2010 [19].

### Benin’s health system and EPI history

Benin’s health system which has a pyramidal structure comprises three different levels [20]. The central level which ensures the implementation of the government-defined health policy is administered by the Ministry of Health. National teaching and specialized hospital facilities are located at this level. The intermediate level is administered by departmental health directions. At this level, health activities are conducted in departmental hospitals, and departmental health directions are in charge of the implementation, coordination of health activities, including epidemiological surveillance. Finally, the peripheral level represents the most decentralized operational entity of the health system. This level is subdivided in health zones. Each zone comprises public and private primary health centers supported by a reference hospital called “zone hospital”.

Initiated in 1982, Benin’s EPI program fights 11 different diseases, including tuberculosis, poliomyelitis, measles, diphtheria, tetanus, pertussis, hepatitis B, *Haemophilus influenzae* type b, pneumococcal disease and rotavirus [21]. The vaccines’ supply chain has also three levels of distribution, including central, intermediate and peripheral levels. The country’s annual vaccine, injection supplies, and needs are estimated by central level teams with technical support from UNICEF. The central level is supplied every six months through UNICEF’s supply chain. Once received, the vaccines are stored in cold rooms at central level, and quarterly distributed to departments by means of refrigerated vehicles. They then deliver to peripheral stores. At peripheral level, two modes of distribution, including both *push* and *pull* approaches coexist. In the *push* approach, vaccines and injection supplies are distributed according to an allocation mode while in the *pull* scenario they are distributed according to a requisition mode. Under the *push* scenario, the distribution of vaccines and injection supplies are monthly supplied by a logistician using a refrigerated vehicle whereas in the *pull* approach health facilities obtain supplies of vaccines and injection supplies on a monthly basis from the health zone to which they belong using motorcycles or private transports. Recently, an optimized push scenario was deployed in selected health zones, with expectation that this optimized logistics system will be the only mode of vaccine distribution at peripheral levels in near future [22].

### Study type and sampling

A retrospective survey was conducted to collect the costs of single- and multi-dose PCV13 using a structured quantitative questionnaire programmed on tablets. A purposive sampling approach which took into account all 3 levels of Bénin’s health pyramid was used to select health structures for data collection. They comprised: 1 structure at central level, 3 departments (Cotonou in the South, Abomey in the Center, and Parakou in the North) at regional level, 17 health zones and 20 primary health centers at district level. This purposive selection of health structures was necessary to ensure availability of the needed data. The study questionnaire is provided as Additional file 1.

### Data collection

The data collection took place from January 1st to February 16th, 2019. Because the multi-dose PCV13 was introduced in April 2018, the study only had data covering a half-year. For this reason, the costs were collected over a period of 6 months, from April 1st to September 30th, 2017 for single-dose PCV13 and from April 1st to September 30, 2018 for multi-dose PCV13. The data collection encompassed various interviews at: central level with the director of the *Agence Nationale de la Vaccination et des Soins de Santé Primaires*; departmental levels with the Chief Medical Officer of the *Service Départemental de la Santé Publique* and of the *Division Vaccination et Recouvrement des Coûts*; peripheral levels with heads of technical and administrative units in charge of vaccines logistics. They aimed at collecting cold chain related costs for both presentation of PCV13’s vaccine, including vaccines, waste management, cold chain, monitoring and supervision, training, human resource, social mobilization, and overheads. These interviews were complemented by data extraction in supporting documents. In total, 16 experienced interviewers were recruited for fieldwork. They received specific training on the use of study instruments before starting the data collection.

### Study perspective, cost estimation and analysis

#### Study perspective

The analysis was conducted from the perspective of the government-funded health service. The economic costs of PCV13 logistics chain before/after the introduction of the multi-dose PCV13 were analyzed. Costs analyzed, including recurrent and capital costs, were identified based on standardized methods [23]. The study questionnaire was based on international developed instruments for costing and financing analyses of routine immunization and new vaccine introduction costs [23]. Furthermore, recurrent and capital costs, including shared and specific costs, were appropriately allocated to each PCV13 presentation. The recurrent costs included vaccines, injection supplies (syringes and safety boxes), human resources (salary and per diems), cold chain, waste management, social mobilization, supervision and monitoring, training, and overheads. The capital costs included training, equipment of cold chain and waste management, and social mobilization. For each PCV13 presentation, total economic costs were obtained by combining expenditure data with input quantities and unit prices.

#### Costs of vaccines

Vaccine costs were estimated as the sum total of vaccine procurement and transportation. Vaccine procurement was obtained by multiplying the unit price per dose by the number of doses dispensed (doses administered plus wasted) over the studied periods for each PCV13 presentation. Unit prices per dose of single- and multi-dose PCV13 were $3.30 and $2.95, respectively [24]. In line with a previous research, transportation costs were estimated at 3% of vaccine procurement values [25].

#### Costs of injection supplies

Costs of syringe and safety boxes were calculated by multiplying quantities used by unit prices of each resource. Quantities used over the studied periods were obtained by summing up quantities of each resource available on April 1st to quantities received during the investigated period minus the remaining on September 30th. Similar to vaccine prices per dose, unit prices of syringes and safety boxes were searched for in UNICEF’s 2018 price list [24].

#### Costs of waste management

Costs of waste management included both recurrent and capital costs incurred for the management of PCV13 wastes. The recurrent costs comprised the costs of training, per diems, maintenance of equipment, fuel for the transportation and incineration of wastes. Recurrent costs that were specific to PCV13 such as specific introductory training and per diems have been fully allocated to the vaccine. Shared costs, including maintenance, fuel, training and per diems for overall vaccination activities were appropriately allocated to each PCV13 presentation in proportion to PCV13 vaccines volume in the cold chain [23]. Capital costs included the economic values of incinerators, discounted at a rate of 3%. Incinerators have been depreciated over 10 years. Since incinerators were shared costs, the estimated half-yearly costs were allocated to PCV13 in proportion to the volume occupied by each PCV13 presentation in the cold chain [23].

#### Costs of cold chain

Costs of cold chain, included recurrent and capital costs incurred for storage and distribution of PCV13 vaccines. The recurrent costs included the costs of storage equipment (ice packs, etc), maintenance of equipment, fuel for transportation, and per diems. Recurrent costs that were specific to PCV13 were fully allocated to the vaccine whereas shared costs were appropriately allocated to each presentation of PCV13 in proportion to the volume of PCV13 vaccines in the cold chain [23]. The half-yearly economic values of capital items discounted at 3%, including cold rooms, refrigerators, voltage regulators, air conditioners, generators, autoclaves, computers, printers, vehicles and motorbikes, have been accounted for in the estimation of cold chain costs. Depreciation of these various capital items followed international recommendations [26], with useful lives of cold rooms, generators, air conditioners, refrigerators and voltage regulators respectively 14, 10, 9, 8 and 6 years. The autoclaves, computers, and printers have been depreciated over 5 years while vehicles and motorbikes were depreciated over 9 and 7 years, respectively. Apart from central level cold room which was fully dedicated to the multi-dose PCV13, the cost of which was entirely allocated to the said vaccine, the half-yearly economic costs of other capital items of the vaccine cold chain were allocated to PCV13 in proportion of the volume occupied by each presentation of PCV13 in the cold chain [23].

#### Costs of monitoring and supervision

The costs of monitoring and supervision took only into account per diems to health personnel for the conduct of monitoring and supervision. Since they were shared costs, they have been thoroughly allocated to each presentation of PCV13 based on recommendations suggested in the literature [23].

#### Costs of training

In addition to the costs of planning for the introduction of the multi-dose PCV13, costs of training, included refresher training for vaccines’ delivery and distribution, record keeping, data management and cold chain maintenance for both presentations of PCV13. Except the costs of training incurred for the introduction of the multi-dose PCV13 which were fully allocated to that vaccine, the costs of other training were allocated to PCV13 in proportion to the volume occupied by each presentation of PCV13 in the cold chain [23]. However, training costs were partial costs since it has not been possible to take into account all costs including experts’ fees and renting of meeting rooms. Training capital costs were depreciated over 2 years.

#### Costs of social mobilization

Social mobilization included the incurred recurrent and capital costs for the conduct of information, education and communication activities. Similar to costs of training, capital costs were amortized over 2 years, discounted at a rate of 3%. Because social mobilizations for the introduction of multi-dose PCV13 were specific costs, they were fully allocated to the vaccine. Social mobilization costs that were not specific have been appropriately allocated to each presentation of PCV13. However, the costs of social mobilization may only have been partial for the single-dose PCV13 since capital items were missing.

#### Costs of human resources

Costs of personnel encompassed the opportunity costs of existing staff. Additional costs of staff recruited due to the introduction of the multi-dose PCV13 were also analyzed for the cited vaccine. The opportunity costs were obtained by multiplying the self-declared weekly working time of each health staff by the corresponding hourly wage. The latter product was multiplied by 28 weeks to obtain the half-yearly estimate of health personnel opportunity costs.

#### Costs of overheads

Overheads included only the recurrent costs for routine functioning of the EPI program. These included stock record forms, delivery and supply forms as well as other miscellaneous expenses. Since these were shared costs, they have been appropriately allocated in proportion to the volume occupied by each presentation of PCV13 in the cold chain [23].

#### Cost analysis

The analysis was conducted in Excel 2010 and Stata 13. Monetary values were reported in US dollars, with US$1 = 559 F CFA (Franc of the Financial Community in Africa) in 2018, and 582 F CFA in 2017 [27]. The total cost of each vaccine presentation was obtained by adding-up half-yearly annualized capital and recurrent costs. However, the capital costs for the single-dose PCV13 were not retrieved during data collection. Because of this, only the capital costs of the multi-dose PCV13 were accounted for. Standard cost aggregation methods were followed to calculate the total cost per PCV13 presentation [23, 28]. Weighted average costs for health centers, zones and departments were multiplied respectively by the total number of health centers, zones, and departments for each presentation of PCV13. Thereafter, the total costs of each vaccine presentation were obtained by adding the estimated costs per level of the health pyramid. Moreover, the cost per infant vaccinated was obtained by dividing the annualized economic costs by the total number of infants vaccinated during the studied period for each vaccine that were recorded during data collection, which were 590,296 and multi- 338,687 for the single-and multi-dose PCV13, respectively. To enable comparison, single-dose PCV13 cost estimates were converted into their 2018 equivalent values, taking inflation into account [29].

## Results

### Annualized, half-yearly economic costs of PCV13

The annualized economic costs of the single-dose PCV13 were slightly higher than the multi-dose PCV13: $3,708,795 Vs $3,698,935 (Table 1). Of the multi-dose PCV13 total costs, annual one-time costs incurred for the introduction of the new vaccine was $308,422, equivalent to 8.34% of the total economic costs of multi-dose PCV13. Furthermore, the economic costs of the single-dose PCV13 were typically constituted of recurrent costs.

**Table 1:** Annualized, half-yearly economic costs of single- and multi-dose PCV13 in Benin (in US$ 2018)

	Single-dose PCV13		Multi-dose PCV13	
Half-yearly annualized	Cost	Percentage	Cost	Percentage
One-time costs	0	0.00	308,422	8.34
Recurrent costs	3,708,796	100.00	3,390,515	91.66
**Total half-yearly annualized costs**	**3,708,796**	**100.00**	**3,698,937**	**100.00**

Independently from the type of PCV13, the recurrent costs were the largest cost component, constituting 100.00 and 91.66% of the single- and multi-dose PCV13 annualized economic costs, respectively (**Table** **2**). Though acquisition of additional capital items occurred, the switch to multi-dose PCV13 seemed to have reduced recurrent costs by $318,282, equivalent to an 8.58% reduction in annualized economic costs. This reduction in costs seemed largely driven by the reduction in recurrent cold chain economic costs, with a decrease of $173,097 i.e. more than half of total reduction of the recurrent costs.

**Table 2:**
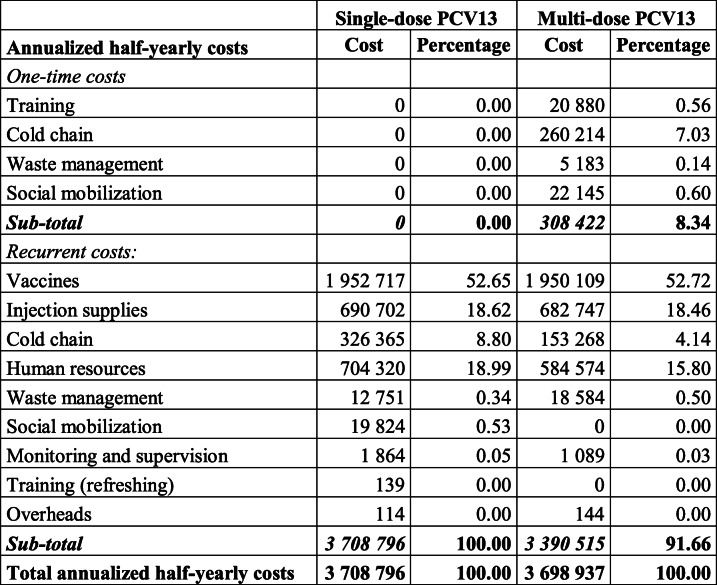
Annualized half-yearly economic costs of PCV13 by type of costs in Benin.

**(in US$ 2018)**

**Table:** 

	Single-dose PCV13		Multi-dose PCV13	
Annualized half-yearly costs	Cost	Percentage	Cost	Percentage
*One-time costs*				
Training	0	0.00	20,880	0.56
Cold chain	0	0.00	260,214	7.03
Waste management	0	0.00	5183	0.14
Social mobilization	0	0.00	22,145	0.60
***Sub-total***	***0***	**0.00**	***308,422***	**8.34**
*Recurrent cost:*				
Vaccines	1,952,717	52.65	1,950,109	52.72
Injection supplies	690,702	18.62	682,747	18.46
Cold chain	326,365	8.80	153,268	4.14
Human resources	704,320	18.99	584,574	15.80
Waste management	12,751	0.34	18,584	0.50
Social mobilization	19,824	0.53	0	0.00
Monitoring and supervision	1864	0.05	1089	0.03
Training (refreshing)	139	0.00	0	0.00
Overheads	114	0.00	144	0.00
***Sub-total***	***3,708,796***	**100.00**	***3,390,515***	**91.66**
**Total annualized half-yearly costs**	**3,708,796**	**100.00**	**3,698,937**	**100.00**

### Annualized, economic costs of PCV13 per infant vaccinated

In spite of the reduction of the total economic costs of the multi-dose PCV13, the cost per infant vaccinated with the single-dose PCV13 was lower than that of the multi-dose: $6.28 Vs $10.92, respectively (Table 3). However, the higher cost per infant vaccinated with the multi-dose PCV13 was largely due to the relatively higher number of infants vaccinated with the single-dose PCV13 compared to multi-dose: 590296 Vs 338,687, respectively.

**Table 3:** Detailed annualized half-yearly economic costs of PCV13 per infant vaccinated in Benin

	Single-dose PCV13		Multi-dose PCV13	
Annualized half-yearly costs	Cost	Percentage	Cost	Percentage
*One-time costs*				
Training	0.00	0.00	0.06	0.56
Cold chain	0.00	0.00	0.77	7.03
Waste management	0.00	0.00	0.02	0.14
Social mobilization	0.00	0.00	0.07	0.60
***Sub-total***	***0.00***	***0.00***	***0.91***	***8.34***
*Recurrent costs*				
Vaccines	3.31	52.65	5.76	52.72
Injection supplies	1.17	18.62	2.02	18.46
Cold chain	0.55	8.80	0.45	4.14
Human resources	1.19	18.99	1.73	15.80
Waste management	0.02	0.34	0.05	0.50
Social mobilization	0.03	0.53	0.00	0.00
Monitoring and supervision	0.00	0.05	0.00	0.03
Training (refreshing)	0.00	0.00	0.00	0.00
Overheads	0.00	0.00	0.00	0.00
***Sub-total***	***6.28***	***100.00***	***10.01***	***91.66***
**Total annualized half-yearly costs**	**6.28**	**100.00**	**10.92**	**100.00**

### Cost of wasted vaccines

The costs of wasted multi-dose PCV13 were higher than that of the single-dose: $1,337,842 Vs $1,236,953, respectively (**Table** **4****)**. The findings also showed a lower cost per vaccine dose wasted for multi-dose PCV13 compared to single-dose PCV13. However, this was not surprising since the number of vaccines wasted was higher for multi-dose PCV13.

**Table 4:** Cost of vaccines wasted before/after introduction of the multi-dose PCV13.

	Single-dose PCV13	Multi-dose PCV13
Total costs of vaccines wasted^b^ (US$ 2018)	1,236,953	1,337,842
Total number of vaccines wasted^c^	355,240	440,297
**Cost per vaccine wasted (US$ 2018)**	**3.48**	**3.04**

^b^Including procurement and freight

^c^Central level estimate

## Discussion

This study brought to light some insights that are worthy of discussion. The findings show that the switch from single- to multi-dose PCV13 has been associated with a reduction in the total costs of PCV13. This reduction seemed to be driven by a decrease in recurrent costs in spite of additional capital costs associated with the introduction of the multi-dose PCV13. Decreases in several recurrent cost components have, jointly, pulled down the total costs of the multi-dose PCV13.

First, the switch from single- to multi-dose PCV13 was associated with a reduction in vaccine cost per dose from $3.3 to $2.95 [24]. This reduction in vaccine cost per dose was also accompanied by the reduction in unit costs of injection supplies [24]. Altogether, these reductions in unit costs of vaccines and injection supplies have contributed to reducing the economic costs of multi-dose PCV13.

Second, the reduction in total costs of multi-dose PCV13 was also associated with a decrease in recurrent cold chain costs. The data analyzed showed a reduction in the volume occupied by the multi-dose PCV13 in the cold chain from 50 to 39%. This finding was in accordance with the results of the logistics research conducted alongside this economic study [22]. It was also in line with a number of previous study findings [30, 31]. This, in turn, may have contributed to lowering recurrent cold chain costs for multi-dose PCV13. This research was unable to retrieve costs data incurred for the introduction of the single-dose PCV13 due in part to the long recall and archiving issues. In spite of the unavailability of the costs incurred for the introduction of the single-dose PCV13, the total cost of the multi-dose PCV13, including the costs incurred for introduction of the latter vaccine, appeared to be lower than that of the single-dose PCV13. This seemed to indicate the switch has globally reduced PCV13 cold chain costs. We believed it to be the case since the annualized economic costs of the single-dose PCV13 were likely underestimated by missing capital costs for training, cold chain, social mobilization and waste management. Besides, previous researches have showed that capital costs incurred for the introduction of the pneumococcal conjugate vaccine (comprising trainings, social mobilization, cold storage, waste management, etc) varied between 4.04 to 4.93% in Gambia [32], Rwanda [33] and Zambia [25]. This means that single-dose PCV13’s total costs may have been much higher if capital costs were available for analysis, and the difference in total costs between the two vaccine presentations may have been much higher.

Third, the reduction in total costs of multi-dose PCV13 seemed also associated with a decrease in total costs of monitoring and supervision. This was surprising since monitoring and supervision associated with the introduction of the multi-dose PCV13 should have been higher for the multi-dose PCV13 compared to that of the single-dose PCV13. However, this could in part be explained by the fact that a number of supervisory activities were not conducted due to budget constraints. Anthropological investigation conducted alongside this economic evaluation reported budget constraints limiting training and supervisory activities of health staff on the multi-dose PCV13 administration [34]. The lack of supervisions, in turn, may have contributed to the increased open vial wasted found in this study. This also was in line with numerous study findings showing significant increases in open vial wasted with increasing vaccine vial size [30, 35, 36]. Our empirical estimations showed high vaccine wastage rates for the multi-dose PCV13 of up to 47% in some facilities. This could contribute, together with additional capital costs, in explaining the observed higher costs of waste management for multi-dose PCV13.

Fourth, the reduction in multi-dose PCV13 total costs is also associated with reduced health personnel costs. This was surprising since the change in vaccine presentation was not associated with changes in human resources involved in cold chain management. However, this finding may be explained by the reduced self-declared staff time devoted to the multi-dose PCV13 cold chain as compared to the single-dose presentation. In turn, reduced human resources’ time invested in the multi-dose PCV13 cold chain may have led to lower human resource costs for the multi-dose PCV13. This is particularly true since the number of infants vaccinated was lower for the multi-dose PCV13 compared to the single-dose PCV13.

Furthermore, the findings showed the costs for training, social mobilization, cold chain and waste management capital costs for the only multi-dose PCV13. These apparent differences in disfavor of the multi-dose PCV13 were only artefact since capital costs for the introduction of the single-dose PCV13 were missing for analysis. Because of this, the total annualized one-time economic costs for the introduction of the single-dose PCV13 may have likely been underestimated compared to figures shown for the multi-dose PCV13. Because of this, we believe that the reduction in annual economic costs associated with the switch to multi-dose PCV13 has been likely underestimated.

The findings also showed a much higher cost per infant vaccinated with the multi-dose PCV13. Unsurprising, this result was associated with the fact that more children were vaccinated with the single-dose PCV13. In line with anthropological research findings on the switch in Benin, this could be explained by the fact that health personnel were not adequately trained on how to administer the multi-dose PCV13, therefore contributing to the observed increased missed vaccine opportunities [37]. For instance, some health personnel refrained to open a multi-dose PCV13 vial when the number of children to be vaccinated was not equal to the number of doses available in the vial. Besides, the present evaluation conducted only six months following the introduction of the multi-dose PCV13 may have also contributed to the high rate of open vial wasted and missed vaccine opportunities via inadequate mastery of the multi-dose PCV13. This finding is corroborated by results of the other logistics and anthropological studies conducted alongside this economic evaluation which found some personnel tending to throw away remaining vaccine doses after vaccination sessions [22, 34]. An improved mastery of the multi-dose PCV13 delivery may have contributed to reducing the cost per infant vaccinated.

Finally, the findings showed a higher total cost of vaccines wasted with the multi-dose PCV13 but with a lower cost per dose of vaccine wasted. This was not surprising given the higher number of vaccines wasted with the multi-dose PCV13. Again, an improved mastery of the multi-dose PCV13 delivery may have contributed to reducing the number of vaccines lost, which, in turn, will lead to reduced total and unit costs of vaccines wasted.

### Limitations

This work has limitations. First, the retrospective approach adopted for data collection may have introduced several biases particularly because of missing data. Missing data is a particular threat in the collection of cost data, especially when record keeping is of poor quality and very inadequate. The analysis revealed a number of missing cost data, including for training, cold chain, social mobilization, waste management, supervision and monitoring, especially for the single-dose PCV13. These may have contributed to distorting the estimates presented in this exercise. In particular, the economic costs of the single-dose PCV13 has likely been underestimated by the fact that capital costs for the introduction of the single-dose PCV13 have not been retrieved and analyzed. This has contributed to the seemingly weak difference in costs between the two presentations of the vaccine. Moreover, central and departmental level personnel participated in train-the-trainer sessions on multi-dose PCV13 administration, with the aim of cascading training within their departments. Owing to budgetary restrictions, cascading training on multi-dose PCV13 were not held. This also may have contributed to distorting the cost estimates.

Second, the purposive selection of study sites may have also distorted the findings because of potential biases. In particular, it introduces selection bias which could have led to under- or over-estimation of costs. Moreover, the health facilities selected as study sites were health facilities with high quality records. This purposive selection could have also biased the findings towards the higher end of performance. Because of this, the findings may not be generalizable.

Third, the estimation of health personnel costs was based on health workers auto-declared changes in their working schedule. An external evaluation, also known as time motion study of health personnel working schedules, would have given better results and limited measurement errors [38].

Finally, this costing assessment was focused on a 6-month data analysis (April to September 2017 and 2018). An annual cost evaluation may have been better since recurrent costs, depending on the level of activity may be subject changes due to seasonal variations in hospital flow. In addition, a longer time-period post-introduction would have been valuable in giving staff appropriate training on the open vial policy which is a critical component of the introduction of the multi-dose vials. Inclusion of adequate training would have increased costs and would potentially have increased the number of multi-dose PCV13 administered. This, in turn, may have contributed to reducing the cost per infant vaccinated.

## Conclusion

This evaluation seemed to show that the switch from single- to multi-dose PCV13 resulted in reduced economic costs of PCV13. This cost reduction, although modest, is globally linked to a decrease in the recurrent costs, including vaccines, injection supplies and cold chain. However, the cost per infant vaccinated was higher with multi-dose PCV13. Therefore, efforts to vaccinate more infants together with a rigorous application of vaccine open vial policy could lead to the change being more cost-effective.

## Supplementary Information

**Additional file 1.** Study questionnaire

## Data Availability

The datasets used and/or analyzed during the current study are available from the corresponding author on reasonable request.

## Abbreviations

BCG: bacillus calmette guerin
DTP: diphtheria tetanus pertussis
EPI: Expanding program for immunization
GAVI: The Vaccine Alliance
GDP: Gross domestic product
MCV: Measles containing vaccine
MoH: Ministry of Health
PCV13: 13-valent pneumococcal vaccine
UNICEF: United Nations International Children’s Emergency Fund
US$: US dollar
WHO: World health organization
