# Supplementary material for: The economic impact of the switch from single- to multi-dose PCV13 vial in Benin
Source: BMC Public Health. 2022 Jan 19;22:133. doi: 10.1186/s12889-021-12108-6 (PMC8772131; doi:10.1186/s12889-021-12108-6)
Supplement: Supplementary file 1 — Additional file 1. Study questionnaire [file 12889_2021_12108_MOESM1_ESM.docx]

**Questionnaire**

This questionnaire aims at evaluating the cost of the switch from the single dose PCV13 (PCV13 SDV) to the multi dose PCV13 (PCV13 MDV). It particularly aims to quantify the resources used for the introduction and implementations of both presentations of the PCV13 vaccine in Benin.

**Caractéristiques générales**

| **N*** | **Question** | **Response code** | **Response** | |
| --- | --- | --- | --- | --- |
| **Q1** | Start date for completing the questionnaire | 🡪 | \|__\|__\|/\|__\|__\|/\|__\|__\|  DD MM YY | |
| **Q2** | Surveyor name | ……………………………………………………………………………………….. | | |
| **Q3** | Surveyed level | Central=1 **🡪 Go to Q8**  Department=2  Health zone=3  Other=9 (Specify  …………………………………………………….) | | \|__\| |
| **Q4** | Name of the department | Cotonou=1  Abomey=2  Parakou=3  Other=9 (Specify  …………………………………………………….) | | \|__\| |
| **Q5** | Name of the health zone | Parakou commune health depot=1  N’Dali commune health depot=2  Abomey zone health depot=3  Bohicon zone health depot=4  Cotonou zone health depot=5  Other=9 (Specify  …………………………………………………….) | | \|__\| |
| **Q6** | Name of the health facility | ……………………………………………………………………………………….. | | |
| **Q8** | What is the qualification of the respondent? | Chief physician=1  Responsible pharmacist =2  Zone manager=3  Head of the health post=4  Administrative officer=5  Other=9 (Specify  …………………………………………………….) | |  |
| **Q9** | At what time does patient care start?  If 7 am, fill in 0700  If 7 pm, fill in 1900 | 1. Monday | |  |
|  |  | 1. Tuesday | |  |
|  |  | 1. Wednesday | |  |
|  |  | 1. Thursday | |  |
|  |  | 1. Friday | |  |
|  |  | 1. Saturday | |  |
|  |  | 1. Sunday | |  |
| **Q10** | At what time does patient care end?  If 7 am, fill in 0700  If 7 pm, fill in 1900 | 1. Monday | |  |
|  |  | 1. Tuesday | |  |
|  |  | 1. Wednesday | |  |
|  |  | 1. Thursday | |  |
|  |  | 1. Friday | |  |
|  |  | 1. Saturday | |  |
|  |  | 1. Sunday | |  |
| **Q11** | At what time do activities / vaccination sessions start?  If 7 am, fill in 0700  If 7 pm, fill in 1900 | 1. Monday | |  |
|  |  | 1. Tuesday | |  |
|  |  | 1. Wednesday | |  |
|  |  | 1. Thursday | |  |
|  |  | 1. Friday | |  |
|  |  | 1. Saturday | |  |
|  |  | 1. Sunday | |  |
| **Q12** | At what time do activities / vaccination sessions end?  If 7 am, fill in 0700  If 7 pm, fill in 1900 | 1. Monday | |  |
|  |  | 1. Tuesday | |  |
|  |  | 1. Wednesday | |  |
|  |  | 1. Thursday | |  |
|  |  | 1. Friday | |  |
|  |  | 1. Saturday | |  |
|  |  | 1. Sunday | |  |
| **Q13** | What is the wastage rate of measles vaccine? | Perrcentage 🡪 | | ……………………..% |

**Vaccines**

This section collects the quantities of PCV13 vaccine administered/used.

**Suveyors** : **For single dose PCV13 (PCV13 SDV) enter the quantities used/consumed from April 1, 2017 to September 30, 2017**

**For multi dose PCV13 (PCV13 MDV) enter the quantities used/consumed from April 1, 2018 to September 30, 2018**

| **Q14. Vaccine’s manufacturer** | **Q.15**  **Doses per vial** | **Q.16**  **Inital stock of vials at the start of the target period**  **(if applicable)** | **Q.17**  **Vials received during the target period** | **Q.18**  **Vials in stock at the end of the target period** | **Q.19**  **Buffer stock (reserve in %)** | **Q.20**  **Doses administered during the target period** | **Q.21**  **How many weeks out of stock during the period have you experienced?** | **Q.22**  **Unit price of a dose** |
| --- | --- | --- | --- | --- | --- | --- | --- | --- |
|  |  |  |  |  |  |  |  |  |
|  |  |  |  |  |  |  |  |  |
|  |  |  |  |  |  |  |  |  |
|  |  |  |  |  |  |  |  |  |
|  |  |  |  |  |  |  |  |  |
|  |  |  |  |  |  |  |  |  |
|  |  |  |  |  |  |  |  |  |
|  |  |  |  |  |  |  |  |  |
|  |  |  |  |  |  |  |  |  |
|  |  |  |  |  |  |  |  |  |

**Syringes, diluents and safety boxes**

This section collects the quantities of syringes and other consumables used for PCV13

**Suveyors** : **For single dose PCV13 (PCV13 SDV) enter the quantities used/consumed from April 1, 2017 to September 30, 2017**

**For multi dose PCV13 (PCV13 MDV) enter the quantities used/consumed from April 1, 2018 to September 30, 2018**

| **Q23. Article** | **Q.24**  **Inital quantity at the start of the target period** | **Q.25 Quantity received during the target period** | **Q.26 Remaining quantity at the end of the target period** | **Q.27**  **Buffer stock (reserve in %)** | **Q.28 Quantity used during the target period** | **Q.29**  **How many weeks out of stock during the period have you experienced?** | **Q.30**  **Unit price** |
| --- | --- | --- | --- | --- | --- | --- | --- |
| Auto-disable syringes |  |  |  |  |  |  |  |
| 10cc syringes |  |  |  |  |  |  |  |
| 5cc syringes |  |  |  |  |  |  |  |
| Reconstitution syringes |  |  |  |  |  |  |  |
| Safety boxes |  |  |  |  |  |  |  |
| Diluent |  |  |  |  |  |  |  |
| Other1(………………………………….) |  |  |  |  |  |  |  |
|  |  |  |  |  |  |  |  |
|  |  |  |  |  |  |  |  |

**Waste management**

**Questions Q31 to Q59 relate to single dose PCV13 (PCV13)**

**Suveyors** : **For single dose PCV13 (PCV13 SDV) enter the quantities used/consumed from April 1, 2017 to September 30, 2017**

| **Q31** | In total, how many incinerators were you using from April 1, 2017 to September 31, 2017 i.e. before the introduction of the PCV13 MDV vaccine? | Response🡪 | ……..……………….……..Number |
| --- | --- | --- | --- |
| **Q32** | Detail on the incinerators used | 1. Brand | ………………….………………………. |
|  |  | 1. Quantity | ………………………………Number |
|  |  | 1. Unit price | ………………………………XOF |
| **Q33** | Were the incinerators listed above only to be used for immunization services? | Yes=1 **🡪Go to Q35**  No=2 | \|__\| |
| **Q34** | If not, on average, what percentage of incinerator use was to be allocated to vaccination? | Response🡪 | ……..……………….……..% |
| **Q35** | Is the incinerator (s) located in the health facility / zone / department or elsewhere? | Inside=1 **🡪Go to Q37**  Elsewhere=2 | \|__\| |
| **Q36** | If elsewhere, how far is the incinerator from the health facility / zone / department? | Distance to the health facility / zone  / department🡪 | ……..……………….……..km |
| **Q37** | How often was the PCV13 SDV waste transported to the incinerator? | 1. Weekly | ………………………………Number |
|  |  | 1. Monthly | ………………………………Number |
|  |  | 1. Quaterly | ………………………………Number |
|  |  | 1. Other (…………………………………….) | ………………………………Number |
| **Q38** | What was the mode of transportation used to transport the waste to the incinerator? | Health facility vehicle =1  Rented vehicle=2  Motorbike =3  Bicycle=4  Other (……………………………….……………….) | \|__\| |
| **Q39** | On average, how much was spent on transporting waste to the incinerator from April 1, 2017 to September 31, 2017 i.e. before the introduction of the PCV13 MDV vaccine? | Amount | ……..……………….……..XOF |
| **Q40** | Were the incinerators installed in a specific room? | Yes=1  No=2 | \|__\| |
| **Q41** | Cost of the room (s) in which the incinerator (s) was (were) before the introduction of PCV13 MDV | Estimated cost of the 1st room | ……..……………….……..XOF |
|  |  | Estimated cost of the 2^nd^ room | ……..……………….……..XOF |
|  |  | Estimated cost of the 3^rd^ room | ……..……………….……..XOF |
| **Q42** | On average, how much was it spent monthly on fuel for operating of the incinerator (s) before the introduction of PCV13 MDV? | Monthly expenditure | ……..……………….……..XOF |
| **Q43** | On average, how much was spend monthly on the maintenance of the incinerator (s) before the introduction of PCV13 MDV? | Monthly expenditure | ……..……………….……..XOF |
| **Q44** | Prior to the introduction of the PCV13 MDV vaccine, have any staff(s) received any training(s) PCV13 SDV waste management? | Yes=1  No=2  Don’t know=3 | \|__\| |
| **Q45** | In total, how many training sessions took place on waste management before the introduction of PCV13 MDV? | Response🡪 | ……..……………….…… Number |
| **Q46** | On average, how many hours per day were spent training individuals on PCV13 waste management and how many days in total did the training last? | Number of hours a day | ……..……………….……. Hours |
|  |  | Number of days | ……..……………….……..Day |
| **Q47** | In total, how much did this (these) training (s) on waste management cost (per diems, room rental, teaching material, coffee break and lunch, etc., included) | Amount of spent over the period | ……..……………….……..XOF |
| **Q48** | In total, how many health workers have been trained on PCV13 SDV waste management prior to the introduction of the PCV13 MDV vaccine | Response🡪 | ……..……………….……..Number |
|  | Please indicate the given names, last names and qualifications of the health personnel who were trained in PCV13 SDV waste management before the introduction of PCV 13 MDV (Insert lines if necessary) | | |
|  | **Gi ven name and last name of the personnel** | **Qualification of the personnel** | **Salary** |
| **Q49** |  |  |  |
| **Q50** |  |  |  |
| **Q51** |  |  |  |
| **Q52** |  |  |  |
| **Q53** |  |  |  |
| **Q54** |  |  |  |
| **Q55** |  |  |  |
| **Q56** |  |  |  |
| **Q57** |  |  |  |
| **Q58** |  |  |  |
| **Q59** |  |  |  |

**Questions Q60 to Q107 relate to multi dose PCV13 (PCV13 MDV)**

**Suveyors** : **For multi dose PCV13 (PCV13 MDV) enter the quantities used/consumed from April 1, 2018 to September 30, 2018**

| **Q60** | Did the introduction of PCV13 MDV require the acquisition of new incinerators in addition to those already existing? | Yes=1  No=2**🡪Go to Q67** | \|__\| |
| --- | --- | --- | --- |
| **Q61** | In total, how many incinerators have you purchased due to the introduction of PCV13 MD? | Response🡪 | ……..……………….……..Number |
| **Q62** | Detail on the incinerators used | 1. Brand | ………………….………………………. |
|  |  | 1. Quantity | ………………………………Number |
|  |  | 1. Unit price | ………………………………XOF |
| **Q63** | Were the incinerators listed above only to be used for immunization services? | Yes=1**🡪Go to Q65**  No=2 | \|__\| |
| **Q64** | If not, on average, what percentage of incinerator use was to be allocated to vaccination? | Response🡪 | ……..……………….……..% |
| **Q65** | Is the incinerator (s) located in the health facility / zone / department or elsewhere? | Inside=1 **🡪Go to Q67**  Elsewhere=2 | \|__\| |
| **Q66** | If elsewhere, how far is the incinerator from the health facility / zone / department? | Distance to the health facility / zone  / department🡪🡪 | ……..……………….……..km |
| **Q67** | How often was the waste transported to the incinerator since the introduction of PCV13 MDV? | 1. Weekly | ………………………………Number |
|  |  | 1. Monthly | ………………………………Number |
|  |  | 1. Quaterly | ………………………………Number |
|  |  | 1. Other (…………………………………….) | ………………………………Number |
| **Q68** | What was the mode of transportation used to transport the waste to the incinerator? | Health facility vehicle =1  Rented vehicle=2  Motorbike =3  Bicycle=4  Other (……………………………….……………….) | \|__\| |
| **Q69** | On average, how much was spent on transporting waste to the incinerator from the introduction of PCV13 MDV vaccine? | Amount | ……..……………….……..XOF |
| **Q70** | Was a room built to house the additional incinerator(s) acquired as a result of the introduction of PCV13 MDV ? | Yes=1  No=2 **🡪Go to Q72** | \|__\| |
| **Q71** | Cost of the room (s) built to house the additional incinerator(s) | Estimated cost of the 1st room | ……..……………….……..XOF |
|  |  | Estimated cost of the 2^nd^ room | ……..……………….……..XOF |
|  |  | Estimated cost of the 3^rd^ room | ……..……………….……..XOF |
| **Q72** | Did the cost of fuel for operating the incenators increased, decreased, or remained the same compared as a result of the PCV13 MDV introduction? | Increased=1 **Go to Q73**  Decreased=2🡪**Go to Q73**  Identical=3**🡪Go to Q74**  Don’t know=4 **🡪Go to Q74** | \|__\| |
| **Q73** | On average in a month, by how much has fuel expenditure increased or decreased since the introduction of PCV13 MDV? | Estimated cost | ……..……………….……..XOF |
| **Q74** | Did the expenditure for incinerator maintenance increased, decreased or remained the same compared to when the PCV13 SDV vaccine was in effect? | Increased=1 **Go to Q75**  Decreased=2🡪**Go to Q75**  Identical=3**🡪Go to Q76**  Don’t know=4 **🡪Go to Q76** | \|__\| |
| **Q75** | On average in a month, by how much did the maintenance expenditure increase or decrease since the introduction of the PCV13 MDV? | Estimated cost | ……..……………….……..XOF |
| **Q76** | To date, have any staff (s) received training(s) on waste management due to introduction of PCV13 MDV? | Yes=1  No=2**🡪Go to Q81** | \|__\| |
| **Q77** | In total, how many training sessions on PCV13 MDV waste management took place ? | Response🡪 | ……..……………….……..Number |
| **Q78** | On average, how many hours per day were spent training individuals on PCV13 MDV waste management and how many days in total did the training last? | Number of hours a day | ……..……………….……..Hours |
|  |  | Number of day of training | ……..……………….……..Days |
| **Q79** | In total, how much did this (these) training (s) on PCV13 MDV waste management cost (per diems, room rental, teaching material, coffee break and lunch, etc., included) | Total cost of training | ……..……………….……..XOF |
| **Q80** | In total, how many health workers have been trained on PCV13 MDV waste management? | Response🡪 | ……..……………….……..Number |
|  | Please indicate the given names, last names and qualifications of the health personnel who were trained in PCV13 MDV waste management (Insert lines if necessary) | | |
|  | **Gi ven name and last name of the personnel** | **Qualification of the personnel** | **Salary** |
| **Q81** |  |  |  |
| **Q82** |  |  |  |
| **Q83** |  |  |  |
| **Q84** |  |  |  |
| **Q85** |  |  |  |
| **Q86** |  |  |  |
| **Q87** |  |  |  |
| **Q88** |  |  |  |
| **Q89** |  |  |  |
| **Q90** |  |  |  |
| **Q91** |  |  |  |
| **Q92** |  |  |  |
| **Q93** |  |  |  |

**Storage and distribution of vaccines**

**Questions Q108 to Q175 relate to single dose PCV13 (PCV13 SDV)**

**Suveyors** : **For single dose PCV13 (PCV13 SDV) enter the quantities used/consumed from April 1, 2017 to September 30, 2017**

| **Q108** | For the management of PCV13 SDV, were cold rooms (s) used for the storage of the vaccines? | Yes=1  No=2 **🡪Go to Q112** | \|__\| |
| --- | --- | --- | --- |
| **Q109** | Please fill in the following sections for the first cold room used for vaccine storage | 1. Total capacity of the cold room in m^2^ | ……..……………….……..m^2^ |
|  |  | 1. Was this cold room only used for PCV13 SDV ?   Yes=1 ; No=2 ; Don’t know=3 | \|__\| |
|  |  | 1. If not, what area of the cold room was used for PCV13 MDV | ……..……………….……..% |
|  |  | 1. Estimated cost of the cold room | ……..……………….……..XOF |
| **Q110** | Please fill in the following sections for the secod cold room used for vaccine storage | 1. Total capacity of the cold room in m^2^ | ……..……………….……..m^2^ |
|  |  | 1. Was this cold room only used for PCV13 SDV ?   Yes=1 ; No=2 ; Don’t know=3 | \|__\| |
|  |  | 1. If not, what area of the cold room was used for PCV13 MDV | ……..……………….……..% |
|  |  | 1. Estimated cost of the cold room | ……..……………….……..XOF |
| **Q111** | Please fill in the following sections for the first cold room used for vaccine storage | 1. Total capacity of the cold room in m^2^ | ……..……………….……..m^2^ |
|  |  | 1. Was this cold room only used for PCV13 SDV ?   Yes=1 ; No=2 ; Don’t know=3 | \|__\| |
|  |  | 1. If not, what area of the cold room was used for PCV13 MDV | ……..……………….……..% |
|  |  | 1. Estimated cost of the cold room | ……..……………….……..XOF |

| **Please fill in the following sections related to costs for maintenance and cold chain from April 1, 2017 to September 31, 2017** | | | | | | | |
| --- | --- | --- | --- | --- | --- | --- | --- |
|  | **Name of equipment** | **Quantity used** | **Brand** | **Make** | **% used for EPI** | **% used for PCV13 SDV** | **Unit price** |
| **Q112** | Hybrid refrigerator |  |  |  |  |  |  |
| **Q113** | Solar refrigerator |  |  |  |  |  |  |
| **Q114** | Thermometer |  |  |  |  |  |  |
| **Q115** | Cooler |  |  |  |  |  |  |
| **Q116** | Insulated boxes |  |  |  |  |  |  |
| **Q117** | Log tag |  |  |  |  |  |  |
| **Q118** | Current regulator |  |  |  |  |  |  |
| **Q119** | Air conditioner |  |  |  |  |  |  |
| **Q120** | Power generator |  |  |  |  |  |  |
| **Q121** | Electric Autoclave |  |  |  |  |  |  |
| **Q122** | Non-electric autoclave |  |  |  |  |  |  |
| **Q123** | Desktop computer |  |  |  |  |  |  |
| **Q124** | Laptop |  |  |  |  |  |  |
| **Q125** | Printer |  |  |  |  |  |  |
| **Q126** | Megaphone |  |  |  |  |  |  |
| **Q127** | Wheelbarrow |  |  |  |  |  |  |
| **Q128** | Cart |  |  |  |  |  |  |
| **Q129** | Devil |  |  |  |  |  |  |
| **Q130** | Deserted plateau |  |  |  |  |  |  |
| **Q131** | Lifting table |  |  |  |  |  |  |
| **Q132** | Winch |  |  |  |  |  |  |
| **Q133** | Table |  |  |  |  |  |  |
| **Q134** | Chair |  |  |  |  |  |  |
| **Q135** | Office |  |  |  |  |  |  |
| **Q136** | Freeze tag |  |  |  |  |  |  |
| **Q137** | Accumulator 0,3l |  |  |  |  |  |  |
| **Q138** | Accumulator 0,4l |  |  |  |  |  |  |
| **Q139** | Accumulator 0,6l |  |  |  |  |  |  |
| **Q140** | Other1(Specify  …………………...............) |  |  |  |  |  |  |
| **Q141** | Other2(Specify  …………………...............) |  |  |  |  |  |  |
| **Q142** | Other3(Specify  …………………...............) |  |  |  |  |  |  |
| **Q143** | Other4(Specify  …………………...............) |  |  |  |  |  |  |
| **Q144** | Other5(Specify  …………………...............) |  |  |  |  |  |  |
| **Q145** | Other5(Specify  …………………...............) |  |  |  |  |  |  |

| **Please fill in the following sections related to costs for maintenance and cold chain from April 1, 2017 to September 31, 2017 PCV13 MDV** | | | |
| --- | --- | --- | --- |
|  | **Question** | **Code of the Response** | **Response** |
| **Q146** | What are the sources of energy used for the cold chain? | 1. Electricity 🡪 | \|___\| Yes=1 No=0 |
|  |  | 1. Gas 🡪 | \|___\| Yes=1 No=0 |
|  |  | 1. Diesel🡪 | \|___\| Yes=1 No=0 |
|  |  | 1. Solar energy 🡪 | \|___\| Yes=1 No=0 |
|  |  | 1. None of the above sources 🡪 | \|___\| Yes=1 No=0 |
| **Q147** | What was the unit price of each energy source used for the cold chain? | 1. Electricity 🡪 | Unit price of a kwh………………………………...XOF |
|  |  | 1. Gas 6kgs 🡪 | Unit price  6kgs ……..………………………...XOF |
|  |  | 1. Gas 12kgs 🡪 | Unit price 12kgs……..……………………….XOF |
|  |  | 1. Diesel 🡪 | Unit price of a liter…..…………………………….XOF |
| **Q148** | On average in a day, how many hours was the cold chain supplied with electricity before the introduction of the PCV13 MDV vaccine? | Number of hours 🡪 | Hours………………………………… |
| **Q149** | On average in a month, how many 6kgs gas cylinders were consumed before the introduction of the PCV13 MDV? | Number of 6kgs gas 🡪 | Number……………………………….. |
| **Q150** | On average in a month, how many 12kgs gas cylinders were consumed before the introduction of the PCV13 MDV? | Number of 12 kgs gas 🡪 | Number………………………………. |
| **Q151** | On average in a month, how many liters of diesel were consumed before the introduction of the PCV13 MDV? | Number of liters of diesel 🡪 | Liters………………………………….. |
| **Q152** | Have you had to install or maintain the solar electrification system in the last 12 months before the introduction of the PCV13 MDV? | Response 🡪 | \|___\| Yes=1 No=0 |
| **Q153** | If yes, how much in total did the installation or maintenance of this solar electrification system cost? | Response 🡪 | Total cost………………………….XOF |

| **Please fill in the following sections related to the rolling stock and maintenance of the rolling stock from April 1, 2017 to September 31, 2017 i.e. before the introduction of PCV13 MDV** | | | | | | | | | | |
| --- | --- | --- | --- | --- | --- | --- | --- | --- | --- | --- |
|  | **Make and model** | **Year of acquisition** | **Total quantity used for the transport of vaccines** | **Additional quantity purchased from April 1, 2017 to September 30, 2017 excluding purchases for the introduction of PCV13 MDV** | **% of the vehicle usage for EPI** | **% used for PCV13 SDV** | **Unit price of the rolling equipment (vehicle, etc)** | **Monthly maintenance expenses of the rolling stock from April 1, 2017 to September 30, 2017 prior to the introduction of PCV 13 MDV** | **Fuel consumption (km / liter)** | **Useful life years** |
| **Q154** |  |  |  |  |  |  |  |  |  |  |
| **Q155** |  |  |  |  |  |  |  |  |  |  |
| **Q156** |  |  |  |  |  |  |  |  |  |  |
| **Q157** |  |  |  |  |  |  |  |  |  |  |
| **Q158** |  |  |  |  |  |  |  |  |  |  |
| **Q159** |  |  |  |  |  |  |  |  |  |  |
| **Q160** |  |  |  |  |  |  |  |  |  |  |
| **Q161** |  |  |  |  |  |  |  |  |  |  |
| **Q162** |  |  |  |  |  |  |  |  |  |  |
| **Q163** |  |  |  |  |  |  |  |  |  |  |
| **Q164** |  |  |  |  |  |  |  |  |  |  |

| **Please complete the following sections related to vaccine collection from April 1, 2017 to September 30, 2017 before the introduction of PCV13 MDV** | | | |
| --- | --- | --- | --- |
|  | **Question** | **Code de la Response** | **Response** |
| **Q169** | What is the distance between your establishment and the vaccine collection point from which you get them? | Distance in kms 🡪 | kms………………………………… |
| **Q170** | How often do you stock up on vaccines? | Once a month=1  Twice a month=2  Once every two months=3  Other (Specify………………………………) | \|___\| |
| **Q171** | What main means of transport did you use to collect / supply the PCV13 SDV vaccines? | Truck from your establishment=1  Car from your establishment =2  Bus trip=3  Motorbike from your establishment =4  Bicycle from your establishment =5  Taxi or rented vehicle=6  Other (Specify ………………………………) | \|___\| |
| **Q172** | What was the cost of round trip transportation for vaccine collection by bus? | Amount in XOF 🡪 | ………………………………… XOF |
| **Q173** | What was the cost of round trip transportation for vaccine collection by taxi or rented vehicle? | Amount in XOF 🡪 | ………………………………… XOF |
| **Q174** | Were per diems paid for the collection of vaccines? | Yes =1 No=2 | \|___\| |
| **Q175** | What is the amount of per diems paid per round trip | Amount in XOF 🡪 | ………………………………… XOF |

**Questions Q176 to Q253 relate to multi dose PCV13 (PCV13 MDV)**

**Suveyors** : **For multi dose PCV13 (PCV13 MDV) enter the quantities used/consumed from April 1, 2018 to September 30, 2018**

| **Q176** | Did the introduction of the PCV13 MDV require the expansion of the storage capacity of the cold room? | Yes=1  No=2 **🡪 Go to Q179a** | \|__\| |
| --- | --- | --- | --- |
| **Q177** | Please fill in the following sections for the additional first cold room | 1. Additional capacity in m^2^ | ……..……………….……..m^2^ |
|  |  | 1. Was this additional cold room only used for the PCV13MDV?   Yes=1 ; No=2 | \|__\| |
|  |  | 1. If not, which cold room surface was used for PCV13 MDV? | ……..……………….……..% |
|  |  | 1. Estimated cost of the cold room | ……..……………….……..XOF |
| **Q178** | Please fill in the following sections for the additional second cold room | 1. Additional capacity in m^2^ | ……..……………….……..m^2^ |
|  |  | 1. Was this additional cold room only used for the PCV13MDV?   Yes=1 ; No=2 | \|__\| |
|  |  | 1. If not, which cold room surface was used for PCV13 MDV? | ……..……………….……..% |
|  |  | 1. Estimated cost of the cold room | ……..……………….……..XOF |
| **Q179** | Please fill in the following sections for the additional third cold room | 1. Additional capacity in m^2^ | ……..……………….……..m^2^ |
|  |  | 1. Was this additional cold room only used for the PCV13MDV?   Yes=1 ; No=2 | \|__\| |
|  |  | 1. If not, which cold room surface was used for PCV13 MDV? | ……..……………….……..% |
|  |  | 1. Estimated cost of the cold room | ……..……………….……..XOF |
| Q179a | If not, has the surface occupied by the PCV13 MDV vaccine remained the same or decreased? | Decreased=1  Identical=2 🡪**Go to Q180** | \|__\| |
| Q179b | If reduced, by how many square meters? | Reduced capacity in m^2^ 🡪 | ……..……………….……..m^2^ |

| **Merci de renseigner les rubriques suivantes relatives aux équipements et fournitures achetés pour l’introduction du PCV13 MDV** | | | | | | | | |
| --- | --- | --- | --- | --- | --- | --- | --- | --- |
|  | **Name of the equipment** | **Total quantity used since the introduction of PCV13 MDV (excluding what was purchased due to the introduction of PCV13MDV)** | **Additional quantity purchased from since the introduction of PCV13 MDV** | **Make** | **Model** | **% of the used for EPI** | **% used for PCV13 MDV** | **Unit price** |
| **Q180** | Hybrid refrigerator |  |  |  |  |  |  |  |
| **Q181** | Solar refrigerator |  |  |  |  |  |  |  |
| **Q182** | Thermometer |  |  |  |  |  |  |  |
| **Q183** | Cooler |  |  |  |  |  |  |  |
| **Q184** | Insulated boxes |  |  |  |  |  |  |  |
| **Q185** | Log tag |  |  |  |  |  |  |  |
| **Q186** | Current regulator |  |  |  |  |  |  |  |
| **Q187** | Air conditioner |  |  |  |  |  |  |  |
| **Q188** | Power generator |  |  |  |  |  |  |  |
| **Q189** | Electric Autoclave |  |  |  |  |  |  |  |
| **Q190** | Non-electric autoclave |  |  |  |  |  |  |  |
| **Q200** | Desktop computer |  |  |  |  |  |  |  |
| **Q201** | Laptop |  |  |  |  |  |  |  |
| **Q202** | Printer |  |  |  |  |  |  |  |
| **Q203** | Megaphone |  |  |  |  |  |  |  |
| **Q204** | Wheelbarrow |  |  |  |  |  |  |  |
| **Q205** | Cart |  |  |  |  |  |  |  |
| **Q206** | Devil |  |  |  |  |  |  |  |
| **Q207** | Deserted plateau |  |  |  |  |  |  |  |
| **Q208** | Lifting table |  |  |  |  |  |  |  |
| **Q209** | Winch |  |  |  |  |  |  |  |
| **Q210** | Table |  |  |  |  |  |  |  |
| **Q211** | Chair |  |  |  |  |  |  |  |
| **Q212** | Office |  |  |  |  |  |  |  |
| **Q213** | Freeze tag |  |  |  |  |  |  |  |
| **Q214** | Accumulator 0,3l |  |  |  |  |  |  |  |
| **Q215** | Accumulator 0,4l |  |  |  |  |  |  |  |
| **Q216** | Accumulator 0,6l |  |  |  |  |  |  |  |
| **Q217** | Other1(Specify  …………………...............) |  |  |  |  |  |  |  |
| **Q218** | Other2(Specify  …………………...............) |  |  |  |  |  |  |  |
| **Q219** | Other3(Specify  …………………...............) |  |  |  |  |  |  |  |
| **Q220** | Other4(Specify  …………………...............) |  |  |  |  |  |  |  |
| **Q221** | Other5(Specify  …………………...............) |  |  |  |  |  |  |  |
| **Q222** | Other6(Specify  …………………...............) |  |  |  |  |  |  |  |

| **Please fill in the following sections related to costs for maintenance and cold chain from April 1, 2018 to September 31, 2018 PCV13 MDV** | | | |
| --- | --- | --- | --- |
|  | **Question** | **Code of the response** | **Response** |
| **Q223** | Did the electricity consumption of the cold chain increased, decreased, or remained the same since the introduction of PCV13 MDV? | Increased=1  Decreased=2  Identical=3 **🡪Go to Q225**  Don’t know=4 **🡪Go to Q225** | \|___\| |
| **Q224** | On average in a month, how many hours has electricity consumption increased or decreased since the introduction of PCV13 MDV? | Number of hours 🡪 | Hours…………………………… |
| **Q225** | Did the monthly consumption of 6kgs gas cylinders for the cold chain increased, decreased, or remained the same since the introduction of PCV13 MDV? | Increased=1  Decreased=2  Identical=3 **🡪Go to Q227**  Don’t know=4 **🡪Go to Q227** | \|___\| |
| **Q226** | On average, of how many the consumption of 6kgs gas cylinders increased or decreased per month? | Number 6kgs gas 🡪 | Quantity………………………… |
| **Q227** | Did the monthly consumption of 12kgs gas cylinders for the cold chain increased, decreased, or remained the same since the introduction of PCV13 MDV? | Increased=1  Decreased=2  Identical=3 **🡪Go to Q229**  Don’t know=4 **🡪Go to Q229** | \|___\| |
| **Q228** | On average, of how many the consumption of 12kgs gas cylinders increased or decreased per month? | Number 12kgs gas 🡪 | Quantity………………………… |
| **Q229** | Did the monthly consumption of diesel for the cold chain increased, decreased, or remained the same since the introduction of PCV13 MDV? | Increased=1  Decreased=2  Identical=3 **🡪Go to Q231**  Don’t know=4 **🡪Go to Q231** | \|___\| |
| **Q230** | On average, of how many the consumption of diesel increased or decreased per month in liters? | Number de liters 🡪 | Liters…………………………… |
| **Q231** | Did you have to install and / or maintain the solar electrification system due to the introduction of the PCV13 MDV? | Yes=1 No=0 **🡪Go to Q233** Response 🡪 | \|___\| |
| **Q232** | If Yes, how much was the total cost of installing and / or maintaining this solar electrification system | Amount in XOF 🡪 | ……………………………XOF |

| **Please fill in the following sections related vehicles and the maintenance of the vehicles used for the storage and distribution of vaccines during the 6 months following the introduction of the PCV13 MDV, i.e. between April 1, 2018 and September 30, 2018** | | | | | | | | |
| --- | --- | --- | --- | --- | --- | --- | --- | --- |
|  | **Make and model** | **Year of acquisition** | **Quantity used since the introduction of PCV13 MDV** | **Additional quantity purchased for the introduction of PCV13 MDV** | **% of the vehicle used for EPI** | **% of the vehicle used PCV13 MDV** | **Unit price** | **Monthly maintenance expenses since the introduction of the PCV13 MDV** |
| **Q233** |  |  |  |  |  |  |  |  |
| **Q234** |  |  |  |  |  |  |  |  |
| **Q235** |  |  |  |  |  |  |  |  |
| **Q236** |  |  |  |  |  |  |  |  |
| **Q237** |  |  |  |  |  |  |  |  |
| **Q238** |  |  |  |  |  |  |  |  |
| **Q239** |  |  |  |  |  |  |  |  |
| **Q240** |  |  |  |  |  |  |  |  |
| **Q241** |  |  |  |  |  |  |  |  |
| **Q242** |  |  |  |  |  |  |  |  |
| **Q243** |  |  |  |  |  |  |  |  |
| **Q244** |  |  |  |  |  |  |  |  |

| **Please complete the following sections related to vaccine collection from April 1, 2018 to September 30, 2018 i.e. since the introduction of PCV13 MDV** | | | |
| --- | --- | --- | --- |
|  | **Question** | **Code of the response** | **Response** |
| **Q245** | What is the distance between your establishment and the vaccine collection point from which you get them? | Distance in kms 🡪 | kms………………………………… |
| **Q246** | Did the frequency of vaccine collection increased, decreased or remained the same since the introduction of the PCV13 MDV vaccine? | Increased=1  Decreased=2  Identical=3 | \|___\| |
| **Q247** | How often do you stock up on vaccines? | Once a month=1  Twice a month=2  Once every two months=3  Other (Specify………………………………) | \|___\| |
| **Q248** | What main means of transport did you use to collect / supply the PCV13 MDV vaccines? | Truck from your establishment=1  Car from your establishment =2  Bus trip=3  Motorbike from your establishment =4  Bicycle from your establishment =5  Taxi or rented vehicle=6  Other (Specify ………………………………) | \|___\| |
| **Q249** | What was the cost of round trip transportation for vaccine collection by bus? | Amount in XOF 🡪 | ………………………………… XOF |
| **Q250** | What was the cost of round trip transportation for vaccine collection by taxi or rented vehicle? | Amount in XOF 🡪 | ………………………………… XOF |
| **Q251** | Were per diems provided for vaccine collection? | Yes =1 No=2 Don’t know=3 | \|___\| |
| **Q252** | Did the amount of per diems paid for vaccine collection increased, decreased or remained the same since the introduction of the PCV13 MDV vaccine? | Increased=1  Decreased=2  Identical=3🡪**Go to Q254**  Don’t know🡪**Go to Q254** | \|___\| |
| **Q253** | By how much the amount of per diems per round trip increases or decreases? | Amount in XOF 🡪 | ………………………………… XOF |

**Surveillance, monitoring and evaluation**

**Questions Q254 to Q260 relate to single dose PCV13 (PCV13 SDV)**

**Suveyors** : **For single dose PCV13 (PCV13 SDV) enter the quantities used/consumed from April 1, 2017 to September 30, 2017**

| **Please fill in the following sections related to the collection of vaccines during the last 12 months preceding the introduction of PCV13 MDV, that is to say between ………………………. and the………………………………** | | | |
| --- | --- | --- | --- |
|  | **Question** | **Code of the response** | **Response** |
| **Q254** | Were the supervision / monitoring visits for vaccines integrated with other supervision /monitoring? | Yes =1 No=2 Don’t know=3 | \|___\| |
| **Q255** | How often were the supervision / monitoring visits carried out by the staff of this establishment before the introduction of the PCV13 MDV? | Once a month=1  Twice a month=2  Once every two month =3  Other (specify………………………………) | \|___\| |
| **Q256** | What means of transport were you using for supervision and monitoring visits before the introduction of the PCV13 MDV? | Truck from your establishment=1  Car from your establishment =2  Bus trip=3  Motorbike from your establishment =4  Bicycle from your establishment =5  Taxi or rented vehicle=6  Other (Specify ………………………………) | \|___\| |
| **Q257** | What was the cost of round trip transportation for supervision and monitoring visit by bus? | Amount in XOF 🡪 | ………………………………… XOF |
| **Q258** | What was the cost of round trip transportation for supervision and monitoring visit by taxi or rented vehicle? | Amount in XOF 🡪 | ………………………………… XOF |
| **Q259** | Were per diems provided to staff for supervision/monitoring before the introduction of PCV13 MDV? | Yes =1 No=2 Don’t know=3 | \|___\| |
| **Q260** | How much was the amount of per diems per supervision/monitoring visit (round trip) | Amount in XOF 🡪 | ………………………………… XOF |

**Questions Q261 to Q275 relate to multi dose PCV13 (PCV13 MDV)**

**Suveyors** : **For multi dose PCV13 (PCV13 MDV) enter the quantities used/consumed from April 1, 2018 to September 30, 2018**

| **Please fill in the following sections related to the collection of vaccines during the 6 months after the introduction of PCV13 MDV, that is to say between ………………………. and the………………………………** | | | |
| --- | --- | --- | --- |
|  | **Question** | **Code of the response** | **Response** |
| **Q261** | Did supervision / monitoring visits for vaccines remained integrated with other supervision /monitoring visits? | Yes =1 No=2 Don’t know=3 | \|___\| |
| **Q262** | Did the frequency of supervision / monitoring visits increased, decreased or remained unchanged since the introduction of PCV13 MDV | Increased=1  Decreased=2  Identical=3  Don’t know =4 | \|___\| |
| **Q263** | How often were the supervision / monitoring visits carried out by the staff of this establishment since the introduction of the PCV13 MDV? | Once a month=1  Twice a month=2  Once every two month =3  Other (specify………………………………) | \|___\| |
| **Q264** | What means of transport were you using for supervision and monitoring visits since the introduction of the PCV13 MDV? | Truck from your establishment=1  Car from your establishment =2  Bus trip=3  Motorbike from your establishment =4  Bicycle from your establishment =5  Taxi or rented vehicle=6  Other (Specify ………………………………) | \|___\| |
| **Q265** | What was the cost of round trip transportation for supervision and monitoring visit by bus since the introduction of PCV13 MDV? | Amount in XOF 🡪 | ………………………………… XOF |
| **Q266** | What was the cost of round trip transportation for supervision and monitoring visit by taxi or rented vehicle since the introduction of PCV13 MDV? | Amount in XOF 🡪 | ………………………………… XOF |
| **Q267** | Did per diems provided to staff for supervision/monitoring changed after the introduction of PCV13 MDV? | Yes =1 No=2 Don’t know=3 | \|___\| |
| **Q268** | How much was the amount of per diems per supervision/monitoring visit since the introduction of PCV13 MDV? | Amount in XOF 🡪 | ………………………………… XOF |

| **Q269** | Did the introduction of the PCV13 MDV vaccine require international travel of personnel to monitor, supervise and evaluate? | Yes =1 No=2 Don’t know=3 | \|___\| |
| --- | --- | --- | --- |
| **Q270** | If Yes, in total how much was spent for the transport of this international staff (plane tickets, buses, etc)? | Amount in XOF 🡪 | ………………………………… XOF |
| **Q271** | If Yes, in total how much was spent in per diems for this international staff? | Amount in XOF 🡪 | ………………………………… XOF |
| **Q273** | Did the introduction of the PCV13 MDV vaccine require travel by ministry of health staff to oversee the implementation of the PCV13 MDV vaccine? | Yes =1 No=2 Don’t know=3 | \|___\| |
| **Q274** | If Yes, in total how much was spent for the transport of the MoH staff (plane tickets, buses, etc)? | Amount in XOF 🡪 | ………………………………… XOF |
| **Q275** | If Yes, in total how much was spent in per diems for the MoH staff? | Amount in XOF 🡪 | ………………………………… XOF |

**Training**

**Questions Q276 to Q281 relate to single dose PCV13 (PCV13 SDV)**

**Suveyors** : **For single dose PCV13 (PCV13 SDV) enter the quantities used/consumed from April 1, 2017 to September 30, 2017**

| **Please fill in the following sections related to training held during the 6 months from April 1, 2017 to September 30, 2017** | | | |
| --- | --- | --- | --- |
|  | **Question** | **Specific question and code of the response** | **Response** |
| **Q276** | Training on traditional EPI vaccines and vaccine distribution | 1. Did training (s) on traditional EPI vaccines and vaccine distribution been held during the 6 months from April 1, 2017 to **September 2017**  ?   Yes =1 No=2 **🡪Go to Q277** Don’t know=3**🡪Go to Q277** | \|___\| |
|  |  | 1. In the past 12 months prior to the introduction of PCV13 MDV, how many staff received training on traditional EPI vaccines and distribution? | Number……………………………. |
|  |  | 1. How many training sessions on traditional EPI vaccines and vaccine distribution have taken place in the last 12 months prior to the introduction of PCV13 MDV? | Number……………………………. |
|  |  | 1. What was the average duration of a training session (in days)? | Day………………………………… |
|  |  | 1. What was the average duration of a training session in terms of hours per day? | Hours……………………………… |
|  |  | 1. How much on average was paid per day in per diems for training? | Amount……………………………XOF |
|  |  | 1. How much did your facility pay for the training on traditional EPI vaccines and vaccine distribution in the last 12 months prior to the introduction of PCV13 MDV? Put 00000 if no expense | Amount……………………………XOF |
|  |  | 1. How much did your facility pay for printing for traditional EPI vaccine training and vaccine distribution in the past 12 months prior to the introduction of PCV13 MDV? Put 00000 if no expense | Amount……………………………XOF |
|  |  | 1. What was the total expenditure made for training on traditional EPI vaccines and vaccine distribution during the last 12 months prior to the introduction of PCV13MDV if details unknown? Put 00000 if no expense. | Amount……………………………XOF |

| **Q277** | Training on record keeping and immunization data management | 1. Was (were) training (s) on record keeping and management of immunization data held during the 6 months from the 1st April 2017 to September 30, 2017?   Yes =1 No=2 **🡪Go to Q278** Don’t know=3**🡪Go to Q278** | \|___\| |
| --- | --- | --- | --- |
|  |  | 1. In the past 12 months prior to the introduction of PCV13 MDV, how many staff received training on record keeping and management of immunization data? | Number……………………………. |
|  |  | 1. How many training sessions on record keeping and management of immunization data have taken place in the last 12 months prior to the introduction of PCV13 MDV? | Number……………………………. |
|  |  | 1. What was the average duration of a training session (in days)? | Day………………………………… |
|  |  | 1. What was the average duration of a training session in terms of hours per day? | Hours……………………………… |
|  |  | 1. How much on average was paid per day in per diems for training? | Amount……………………………XOF |
|  |  | 1. How much did your facility pay for the training on record keeping and management of immunization data in the last 12 months prior to the introduction of PCV13 MDV? Put 00000 if no expense | Amount……………………………XOF |
|  |  | 1. How much did your facility pay for printing for record keeping and management of immunization data training in the past 12 months prior to the introduction of PCV13 MDV? Put 00000 if no expense | Amount……………………………XOF |
|  |  | 1. What was the total expenditure made for training on record keeping and management of immunization data during the last 12 months prior to the introduction of PCV13MDV if details unknown? Put 00000 if no expense. | Amount……………………………XOF |

| **Q278** | Training on the maintenance of the vaccine cold chain | 1. Was (were) training (s) on the maintenance of the vaccine cold chain held during the 6 months from April 1, 2017 to April 30 September 2017?   Yes =1 No=2 **🡪Go to Q279** Don’t know=3**🡪Go to Q279** | \|___\| |
| --- | --- | --- | --- |
|  |  | 1. In the past 12 months prior to the introduction of PCV13 MDV, how many staff received training on the maintenance of the vaccine cold chain? | Number……………………………. |
|  |  | 1. How many training sessions on the maintenance of the vaccine cold chain have taken place in the last 12 months prior to the introduction of PCV13 MDV? | Number……………………………. |
|  |  | 1. What was the average duration of a training session (in days)? | Day………………………………… |
|  |  | 1. What was the average duration of a training session in terms of hours per day? | Hours……………………………… |
|  |  | 1. How much on average was paid per day in per diems for training? | Amount……………………………XOF |
|  |  | 1. How much did your facility pay for the training on maintenance of the vaccine cold chain in the last 12 months prior to the introduction of PCV13 MDV? Put 00000 if no expense | Amount……………………………XOF |
|  |  | 1. How much did your facility pay for printing for maintenance of the vaccine cold chain training in the past 12 months prior to the introduction of PCV13 MDV? Put 00000 if no expense | Amount……………………………XOF |
|  |  | 1. What was the total expenditure made for training on maintenance of the vaccine cold chain during the last 12 months prior to the introduction of PCV13MDV if details unknown? Put 00000 if no expense. | Amount……………………………XOF |

| **Q279** | Training on management of stock of vaccines and delivery | 1. Was (were) training (s) on management of stock of vaccines and delivery held during the 6 months from April 1, 2017 to April 30 September 2017?   Yes =1 No=2 **🡪Go to Q280** Don’t know=3**🡪Go to Q280** | \|___\| |
| --- | --- | --- | --- |
|  |  | b. In the past 12 months prior to the introduction of PCV13 MDV, how many staff received training on management of stock of vaccines and delivery? | Number……………………………. |
|  |  | c. How many training sessions on management of stock of vaccines and delivery have taken place in the last 12 months prior to the introduction of PCV13 MDV? | Number……………………………. |
|  |  | d. What was the average duration of a training session (in days)? | Day………………………………… |
|  |  | e. What was the average duration of a training session in terms of hours per day? | Hours……………………………… |
|  |  | f. How much on average was paid per day in per diems for training? | Amount……………………………XOF |
|  |  | g. How much did your facility pay for the training on management of stock of vaccines and delivery in the last 12 months prior to the introduction of PCV13 MDV? Put 00000 if no expense | Amount……………………………XOF |
|  |  | h. How much did your facility pay for printing for the management of stock of vaccines and delivery training in the past 12 months prior to the introduction of PCV13 MDV? Put 00000 if no expense | Amount……………………………XOF |
|  |  | i. What was the total expenditure made for training on management of stock of vaccines and delivery during the last 12 months prior to the introduction of PCV13MDV if details unknown? Put 00000 if no expense. | Amount……………………………XOF |

| **Q280** | Training on immunization planning and management | 1. Was (were) training (s) on immunization planning and management held during the 6 months from April 1, 2017 to April 30 September 2017?   Yes =1 No=2 **🡪Go to Q281** Don’t know=3**🡪Go to Q281** | \|___\| |
| --- | --- | --- | --- |
|  |  | 1. In the past 12 months prior to the introduction of PCV13 MDV, how many staff received training on immunization planning and management? | Number……………………………. |
|  |  | 1. How many training sessions on immunization planning and management have taken place in the last 12 months prior to the introduction of PCV13 MDV? | Number……………………………. |
|  |  | 1. What was the average duration of a training session (in days)? | Day………………………………… |
|  |  | 1. What was the average duration of a training session in terms of hours per day? | Hours……………………………… |
|  |  | 1. How much on average was paid per day in per diems for training? | Amount……………………………XOF |
|  |  | 1. How much did your facility pay for the training on immunization planning and management in the last 12 months prior to the introduction of PCV13 MDV? Put 00000 if no expense | Amount……………………………XOF |
|  |  | 1. How much did your facility pay for printing for immunization planning and management training in the past 12 months prior to the introduction of PCV13 MDV? Put 00000 if no expense | Amount……………………………XOF |
|  |  | 1. What was the total expenditure made for training on immunization planning and management during the last 12 months prior to the introduction of PCV13MDV if details unknown? Put 00000 if no expense. | Amount……………………………XOF |

| **Q281** | Training on the introduction of the new PCV13 MDV vaccine | 1. Was (were) training (s) on the introduction of the new PCV13 MDV vaccine held during the 6 months from April 1, 2017 to April 30 September 2017?   Yes =1 No=2 **🡪Go to Q282** Don’t know=3**🡪Go to Q282** | \|___\| |
| --- | --- | --- | --- |
|  |  | 1. In the past 12 months prior to the introduction of PCV13 MDV, how many staff received training on the introduction of the new PCV13 MDV vaccine? | Number……………………………. |
|  |  | 1. How many training sessions on the introduction of the new PCV13 MDV vaccine have taken place in the last 12 months prior to the introduction of PCV13 MDV? | Number……………………………. |
|  |  | 1. What was the average duration of a training session (in days)? | Day………………………………… |
|  |  | 1. What was the average duration of a training session in terms of hours per day? | Hours……………………………… |
|  |  | 1. How much on average was paid per day in per diems for training? | Amount……………………………XOF |
|  |  | 1. How much did your facility pay for the training on the introduction of the new PCV13 MDV vaccine in the last 12 months? Put 00000 if no expense | Amount……………………………XOF |
|  |  | 1. How much did your facility pay for printing for the introduction of the new PCV13 MDV vaccine training in the past 12 months? Put 00000 if no expense | Amount……………………………XOF |
|  |  | 1. What was the total expenditure made for training on the introduction of the new PCV13 MDV vaccine during the last 12 months? Put 00000 if no expense. | Amount……………………………XOF |

**Questions Q282 to Q287 relate to training sessions held**

**Suveyors** : **For multi dose PCV13 (PCV13 MDV) enter the quantities used/consumed from April 1, 2018 to September 30, 2018**

| **Please fill in the following sections related to training held from April 1, 2018 to September 30, 2018** | | | |
| --- | --- | --- | --- |
|  | **Question** | **Specific question and code of the response** | **Response** |
| **Q282** | Refresher training on vaccine distribution? | 1. Was (were) refresher training on PCV13 MDV vaccine distribution held during the 6 months from April 1, 2018 to April 30 September 2018?   Yes =1 No=2 **🡪Go to Q283** Don’t know=3**🡪Go to Q283** | \|___\| |
|  |  | 1. In the 6 months following the introduction of PCV13 MDV, how many staff received refresher training on vaccine distribution? | Number……………………………. |
|  |  | 1. How many refresher training sessions on vaccine distribution have taken place in the 6 months following the introduction of PCV13 MDV? | Number……………………………. |
|  |  | 1. What was the average duration of a training session (in days)? | Day………………………………… |
|  |  | 1. What was the average duration of a training session in terms of hours per day? | Hours……………………………… |
|  |  | 1. How much on average was paid per day in per diems for training? | Amount……………………………XOF |
|  |  | 1. How much did your facility pay for the refresher training sessions on vaccine distribution in the last 6 months following the introduction of PCV13MDV? Put 00000 if no expense | Amount……………………………XOF |
|  |  | 1. How much did your facility pay for printing for the refresher training sessions on vaccine distribution in the 6 months following the introduction of PCV13MDV? Put 00000 if no expense | Amount……………………………XOF |
|  |  | 1. What was the total expenditure made for training on the refresher training sessions on vaccine distribution during the 6 months following the introduction of PCV13MDV? Put 00000 if no expense. | Amount……………………………XOF |

| **Q283** | Refresher training on record keeping and immunization data management | 1. Was (were) refresher training (s) on record keeping and management of immunization data held during the 6 months from the 1st April 2018 to September 30, 2018?   Yes =1 No=2 **🡪Go to Q284** Don’t know=3**🡪Go to Q284** | \|___\| |
| --- | --- | --- | --- |
|  |  | 1. In the 6 months following the introduction of PCV13 MDV, how many staff received refresher training on record keeping and management of immunization data? | Number……………………………. |
|  |  | 1. How many refresher training sessions on record keeping and management of immunization data have taken place in the 6 months following the introduction of PCV13 MDV? | Number……………………………. |
|  |  | 1. What was the average duration of a training session (in days)? | Day………………………………… |
|  |  | 1. What was the average duration of a training session in terms of hours per day? | Hours……………………………… |
|  |  | 1. How much on average was paid per day in per diems for training? | Amount……………………………XOF |
|  |  | 1. How much did your facility pay for refresher training on record keeping and management of immunization data in the 6 months following the introduction of PCV13 MDV? Put 00000 if no expense | Amount……………………………XOF |
|  |  | 1. How much did your facility pay for printing for refresher record keeping and management of immunization data training in the 6 months following the introduction of PCV13 MDV? Put 00000 if no expense | Amount……………………………XOF |
|  |  | 1. What was the total expenditure made for training on record keeping and management of immunization data during the 6 months following the introduction of PCV13MDV? Put 00000 if no expense. | Amount……………………………XOF |

| **Q284** | Refresher training on the maintenance of the vaccine cold chain | 1. Was (were) refresher training (s) on the maintenance of the vaccine cold chain held during the 6 months from April 1, 2018 to April 30 September 2018?   Yes =1 No=2 **🡪Go to Q285** Don’t know=3**🡪Go to Q285** | \|___\| |
| --- | --- | --- | --- |
|  |  | 1. In the 6 months following the introduction of PCV13 MDV, how many staff received refresher training on the maintenance of the vaccine cold chain? | Number……………………………. |
|  |  | 1. How many refresher training sessions on the maintenance of the vaccine cold chain have taken place in the 6 months following the introduction of PCV13 MDV? | Number……………………………. |
|  |  | 1. What was the average duration of a training session (in days)? | Day………………………………… |
|  |  | 1. What was the average duration of a training session in terms of hours per day? | Hours……………………………… |
|  |  | 1. How much on average was paid per day in per diems for training? | Amount……………………………XOF |
|  |  | 1. How much did your facility pay for the refresher training on maintenance of the vaccine cold chain in the 6 months following the introduction of PCV13 MDV? Put 00000 if no expense | Amount……………………………XOF |
|  |  | 1. How much did your facility pay for printing for refresher training on maintenance of the vaccine cold chain in the 6 months following the introduction of PCV13 MDV? Put 00000 if no expense | Amount……………………………XOF |
|  |  | 1. What was the total expenditure made for refresher training on maintenance of the vaccine cold chain during the 6 months following the introduction of PCV13MDV? Put 00000 if no expense. | Amount……………………………XOF |

| **Q285** | Refresher training on management of stock of vaccines and delivery | 1. Was (were) refresher training (s) on management of stock of vaccines and delivery held   during the 6 months from April 1, 2018 to April 30 September 2018?  Yes =1 No=2 **🡪Go to Q286** Don’t know=3**🡪Go to Q286** | \|___\| |
| --- | --- | --- | --- |
|  |  | 1. In the 6 months following the introduction of PCV13 MDV, how many staff received   refresher training on management of stock of vaccines and delivery? | Number……………………………. |
|  |  | 1. How many refresher training sessions on management of stock of vaccines and delivery   have taken place in the 6 months following the introduction of PCV13 MDV? | Number……………………………. |
|  |  | d. What was the average duration of a training session (in days)? | Day………………………………… |
|  |  | e. What was the average duration of a training session in terms of hours per day? | Hours……………………………… |
|  |  | f. How much on average was paid per day in per diems for training? | Amount……………………………XOF |
|  |  | 1. How much did your facility pay for the refresher training on management of stock of   vaccines and delivery in the 6 months following the introduction of PCV13 MDV? Put 00000  if no expense | Amount……………………………XOF |
|  |  | 1. How much did your facility pay for printing for the management of stock of vaccines and   delivery refresher training in the 6 months following the introduction of PCV13 MDV? Put 00000 if no expense | Amount……………………………XOF |
|  |  | 1. What was the total expenditure made for the refresher training on management of stock of vaccines and delivery during the 6 months following the introduction of PCV13MDV? Put 00000 if no expense. | Amount……………………………XOF |

| **Q286** | Refresher training on immunization planning and management | 1. Was (were) refresher training (s) on immunization planning and management held during the 6 months from April 1, 2018 to April 30 September 2018?   Yes =1 No=2 **🡪Go to Q287** Don’t know=3**🡪Go to Q287** | \|___\| |
| --- | --- | --- | --- |
|  |  | 1. In the 6 months following the introduction of PCV13 MDV, how many staff received refresher training on immunization planning and management? | Number……………………………. |
|  |  | 1. How many training sessions on immunization planning and management have taken place   in the 6 months following the introduction of PCV13 MDV? | Number……………………………. |
|  |  | 1. What was the average duration of a training session (in days)? | Day………………………………… |
|  |  | 1. What was the average duration of a training session in terms of hours per day? | Hours……………………………… |
|  |  | 1. How much on average was paid per day in per diems for training? | Amount……………………………XOF |
|  |  | 1. How much did your facility pay for the refresher training on immunization planning and   management in the 6 months follwing the introduction of PCV13 MDV? Put 00000 if no  expense | Amount……………………………XOF |
|  |  | 1. How much did your facility pay for printing for immunization planning and management   refresher training in the 6 months following the introduction of PCV13 MDV? Put 00000 if no expense | Amount……………………………XOF |
|  |  | 1. What was the total expenditure made for refresher training on immunization planning and   management during the 6 months following the introduction of PCV13MDV? Put 00000 if no expense. | Amount……………………………XOF |

| **Q287** | Refresher training on the introduction of the new PCV13 MDV vaccine | 1. Was (were) refresher training (s) on the introduction of the new PCV13 MDV vaccine held during the 6 months from April 1, 2018 to April 30 September 2018?   Yes =1 No=2 **🡪Go to Q288** Don’t know=3**🡪Go to Q288** | \|___\| |
| --- | --- | --- | --- |
|  |  | 1. In the 6 months following the introduction of PCV13 MDV, how many staff received refresher training on the introduction of the new PCV13 MDV vaccine? | Number……………………………. |
|  |  | 1. How many refresher training sessions on the introduction of the new PCV13 MDV vaccine have taken place in the 6 months following the introduction of PCV13 MDV? | Number……………………………. |
|  |  | 1. What was the average duration of a training session (in days)? | Day………………………………… |
|  |  | 1. What was the average duration of a training session in terms of hours per day? | Hours……………………………… |
|  |  | 1. How much on average was paid per day in per diems for training? | Amount……………………………XOF |
|  |  | 1. How much did your facility pay for the refresher training on the introduction of the new PCV13 MDV vaccine in the 6 months following the introduction of PCV13 MDV? Put 00000 if no expense | Amount……………………………XOF |
|  |  | 1. How much did your facility pay for printing for the introduction of the new PCV13 MDV vaccine training in the 6 months? Put 00000 if no expense | Amount……………………………XOF |
|  |  | 1. What was the total expenditure made for training on the introduction of the new PCV13 MDV vaccine during the last 6 months? Put 00000 if no expense. | Amount……………………………XOF |

**Social mobilization**

**Questions Q288 to Q294 relate to activities for social mobilization held in the last 12 months prior to the introduction of PCV13 MDV**

| **Please fill in the following sections related to social mobilization activities held during the last 12 months preceding the introduction of PCV13 MDV** | | | |
| --- | --- | --- | --- |
|  | **Question** | **Code of the response** | **Response** |
| **Q288** | In the past 12 months prior to the introduction of PCV13 MDV, how many social mobilization events on vaccination have been organized? | Response 🡪 | Number ………………………………… |
| **Q289** | Did the organization of these social mobilization events require travels? | Yes =1 No=2 Don’t know=3 | \|___\| |
| **Q290** | If yes, what was the average distance traveled for each social mobilization event? | Distance in kms 🡪 | ………………………………… kms |
| **Q291** | On average, how many health workers in your facility were involved in each event? | Response 🡪 | Number ………………………………… |
| **Q292** | On average, how many days of per diems were allocated for each social mobilization event? | Response 🡪 | Day….. ………………………… |
| **Q293** | What is the amount of per diems paid per day per staff involved? | Amount in XOF 🡪 | ………………………………… XOF |
| **Q293** | How much was spent on the production of materials for these events including radio spots (excluding per diems) | Amount in XOF 🡪 | ………………………………… XOF |
| **Q294** | Other expense…………………………………………………………… | Amount in XOF 🡪 | ………………………………… XOF |

**Questions Q295 to Q302 relate to activities for social mobilization held in the 6 months following the introduction of PCV13 MDV**

| **Please fill in the following sections related to social mobilization activities held during the 6 months following the introduction of PCV13 MDV** | | | |
| --- | --- | --- | --- |
|  | **Question** | **Code of the response** | **Response** |
| **Q295** | In the 6 months following the introduction of PCV13 MDV, how many social mobilization events on vaccination have been organized? | Response 🡪 | Number ………………………………… |
| **Q296** | Did the organization of these social mobilization events require travels? | Yes =1 No=2 Don’t know=3 | \|___\| |
| **Q297** | If yes, what was the average distance traveled for each social mobilization event? | Distance in kms 🡪 | ………………………………… kms |
| **Q298** | On average, how many health workers in your facility were involved in each event? | Response 🡪 | Number ………………………………… |
| **Q299** | On average, how many days of per diems were allocated for each social mobilization event? | Response 🡪 | Day….. ………………………… |
| **Q300** | What is the amount of per diems paid per day per staff involved? | Amount in XOF 🡪 | ………………………………… XOF |
| **Q301** | How much was spent on the production of materials for these events including radio spots (excluding per diems) | Amount in XOF 🡪 | ………………………………… XOF |
| **Q302** | Other expense…………………………………………………………… | Amount in XOF 🡪 | ………………………………… XOF |

**Others additional costs**

**Questions Q303 to Q311 relate to other additional costs borne during the 12 months preceeding the introduction of PCV13 MDV**

| **Please fill in the following sessions on the operational costs borne during the last 12 months preceding the introduction of the PCV13 MDV** | | | |
| --- | --- | --- | --- |
|  | **Question** | **Quantity used** | **Unit price** |
| **Q303** | Order registry | …………………………………Number | ………………………………… XOF |
| **Q304** | Order form | …………………………………Number | ………………………………… XOF |
| **Q305** | Purchase order | …………………………………Number | ………………………………… XOF |
| **Q306** | Delivery form | …………………………………Number | ………………………………… XOF |
| **Q307** | Vaccination card | …………………………………Number | ………………………………… XOF |
| **Q308** | Other1 (specify………………………………………………………….) | …………………………………Number | ………………………………… XOF |
| **Q309** | Other2 (specify………………………………………………………….) | …………………………………Number | ………………………………… XOF |
| **Q310** | Other3 (specify………………………………………………………….) | …………………………………Number | ………………………………… XOF |
| **Q311** | Other4 (specify………………………………………………………….) | …………………………………Number | ………………………………… XOF |

**Questions Q312 to Q320 relate to other additional costs borne during the 6 months following the introduction of PCV13 MDV**

| **Please fill in the following sessions on the operational costs borne during the 6 months following the introduction of the PCV13 MDV** | | | |
| --- | --- | --- | --- |
|  | **Question** | **Quantity used** | **Unit price** |
| **Q312** | Order registry | …………………………………Number | ………………………………… XOF |
| **Q313** | Order form | …………………………………Number | ………………………………… XOF |
| **Q314** | Purchase order | …………………………………Number | ………………………………… XOF |
| **Q315** | Delivery form | …………………………………Number | ………………………………… XOF |
| **Q316** | Vaccination card | …………………………………Number | ………………………………… XOF |
| **Q317** | Other1 (specify………………………………………………………….) | …………………………………Number | ………………………………… XOF |
| **Q318** | Other2 (specify………………………………………………………….) | …………………………………Number | ………………………………… XOF |
| **Q319** | Other3 (specify………………………………………………………….) | …………………………………Number | ………………………………… XOF |
| **Q320** | Other4 (specify………………………………………………………….) | …………………………………Number | ………………………………… XOF |

**Personnel**

**Permanent staff of the structure**

|  | **Interviewer: List all the staff involved on immunization activities. (give the staff's qualification; when several people have the same qualification, write down [Qualif 1], [Qualif 2], etc.)** | **Has this staff been specially recruited for activities linked only to the PCV13 vaccine MDV?**  **(Yes=1 No=0)** | **How many hours per week did this staff usually spend on immunization activities before the introduction of the PCV13 MDV vaccine** | **Out of the time spent on immunization activities, which percentage was devoted to PCV13 SDV** | **Did the weekly time devoted to PCV13 increase since the introduction of the PCV13 MDV vaccine**  **(Yes=1 No=0)** | **If Yes, how many hours per week has this working time increased since the introduction of the PCV13 MDV** | **What is the employee's gross monthly salary?** |
| --- | --- | --- | --- | --- | --- | --- | --- |
| **Q321** |  |  |  |  |  |  |  |
| **Q322** |  |  |  |  |  |  |  |
| **Q323** |  |  |  |  |  |  |  |
| **Q323** |  |  |  |  |  |  |  |
| **Q325** |  |  |  |  |  |  |  |
| **Q326** |  |  |  |  |  |  |  |
| **Q327** |  |  |  |  |  |  |  |
| **Q328** |  |  |  |  |  |  |  |
| **Q329** |  |  |  |  |  |  |  |
| **Q330** |  |  |  |  |  |  |  |
| **Q331** |  |  |  |  |  |  |  |
| **Q332** |  |  |  |  |  |  |  |
| **Q333** |  |  |  |  |  |  |  |

**Permanent staff (community health workers) of the structure**

|  | **Interviewer: List all the staff involved on immunization activities. (give the staff's qualification; when several people have the same qualification, write down [Qualif 1], [Qualif 2], etc.)** | **Has this staff been specially recruited for activities linked only to the PCV13 vaccine MDV?**  **(Yes=1 No=0)** | **How many hours per week did this staff usually spend on immunization activities before the introduction of the PCV13 MDV vaccine** | **Out of the time spent on immunization activities, which percentage was devoted to PCV13 SDV** | **Did the weekly time devoted to PCV13 increase since the introduction of the PCV13 MDV vaccine**  **(Yes=1 No=0)** | **If Yes, how many hours per week has this working time increased since the introduction of the PCV13 MDV** | **What is the employee's gross monthly salary?** |
| --- | --- | --- | --- | --- | --- | --- | --- |
| **Q334** |  |  |  |  |  |  |  |
| **Q335** |  |  |  |  |  |  |  |
| **Q336** |  |  |  |  |  |  |  |
| **Q337** |  |  |  |  |  |  |  |
| **Q338** |  |  |  |  |  |  |  |
| **Q339** |  |  |  |  |  |  |  |
| **Q340** |  |  |  |  |  |  |  |
| **Q341** |  |  |  |  |  |  |  |
| **Q342** |  |  |  |  |  |  |  |
| **Q343** |  |  |  |  |  |  |  |
| **Q344** |  |  |  |  |  |  |  |
| **Q345** |  |  |  |  |  |  |  |
| **Q346** |  |  |  |  |  |  |  |

**Thanks !**
